# Mucinous adenocarcinoma of the appendix presenting as acute appendicitis: a case report

**DOI:** 10.1093/jscr/rjae713

**Published:** 2024-11-18

**Authors:** Asham Al Salkhadi, Mohammad Ajwad Al Salkhadi, Ayham Hasan

**Affiliations:** Department of General Surgery, Mubarak Al Kabeer Hospital, Al-Jabriyah 32001, Kuwait; Department of Radiology, Jordan University of Science and Technology, Irbid 22110, Jordan; Faculty of Medicine, Tishreen University, Lattakia 041, Syria

**Keywords:** mucinous, adenocarcinoma, appendix, appendiceal cancer, MAA

## Abstract

Mucinous adenocarcinoma of the appendix (MAA) is a rare primary malignancy with an incidence of 0.01–0.2% and often presents as acute appendicitis. We present a case of a 61-year-old male who initially presented with right iliac fossa pain, worsening over 3 days, accompanied by appetite loss but no other symptoms. The patient’s history included a splenectomy and epilepsy. Physical examination and computed tomography scan suggested a picture of acute appendicitis. He underwent an emergency laparoscopic appendectomy that was converted to open right hemicolectomy due to the mass’s adherence to the cecum. The mass, at the cecum, originating from the appendix, measured 10 × 7 × 7 cm. Pathology confirmed a moderately differentiated MAA. Accurate diagnosis requires a combination of imaging and histopathology. The patient recovered well and was discharged on Day 6 postoperatively. We aim to highlight the importance of distinguishing MAA from acute appendicitis and the need for careful preoperative evaluation.

## Introduction

Primary appendiceal neoplasms are rare and found in 1% of appendectomy specimens [1]. Two-thirds of these cases are adenocarcinomas, with mucinous adenocarcinoma of the appendix (MAA) being the most common subtype [2]. MAA accounts for 0.01–0.2% of gastrointestinal tumors and typically presents around the age of 60 [3] and often mimics acute appendicitis. Diagnosis requires a combination of computed tomography (CT) imaging and histopathology. We present the case of a 61-year-old male initially suspected of having acute appendicitis, which was later confirmed as MAA after surgical resection and histopathological sampling.

## Case report

A 61-year-old male presented to the emergency department with sharp, constant pain in the right iliac fossa that began 3 days ago and kept progressively worsening. The pain was associated with a loss of appetite but no nausea, vomiting, fever, or bowel changes. His history included a splenectomy 38 years ago, and epilepsy treated with valproic acid. On examination, McBurney’s sign and rebound tenderness were positive. Blood tests showed elevated white blood cells (16.60 10^3^/μl; normal range: 3.7–10), creatinine (139 μmol/l; normal range: 64–104), and urea (9.7; mmol/l; normal range: 2.8–7.2).

**Figure 1:**
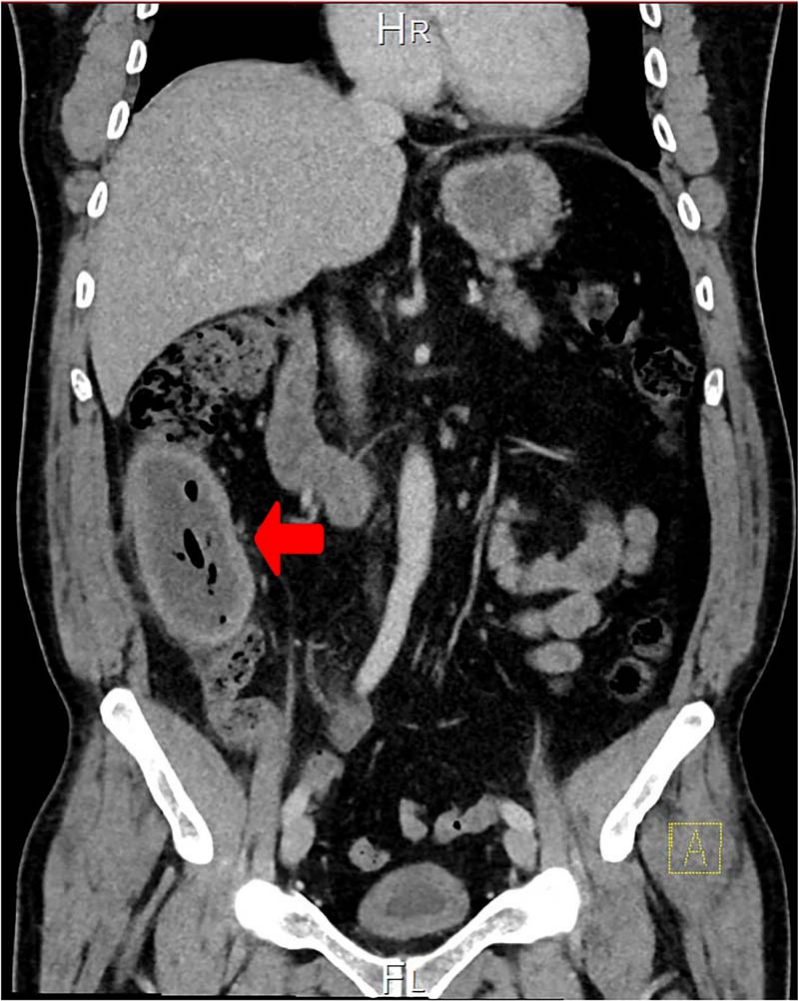
A coronal CT view reveals an oval mass in the right iliac fossa. The mass has a thick, edematous wall and contains fluid and gas. Extensive fat stranding is visible, but no free fluid or bowel dilation is noted.

**Figure 2:**
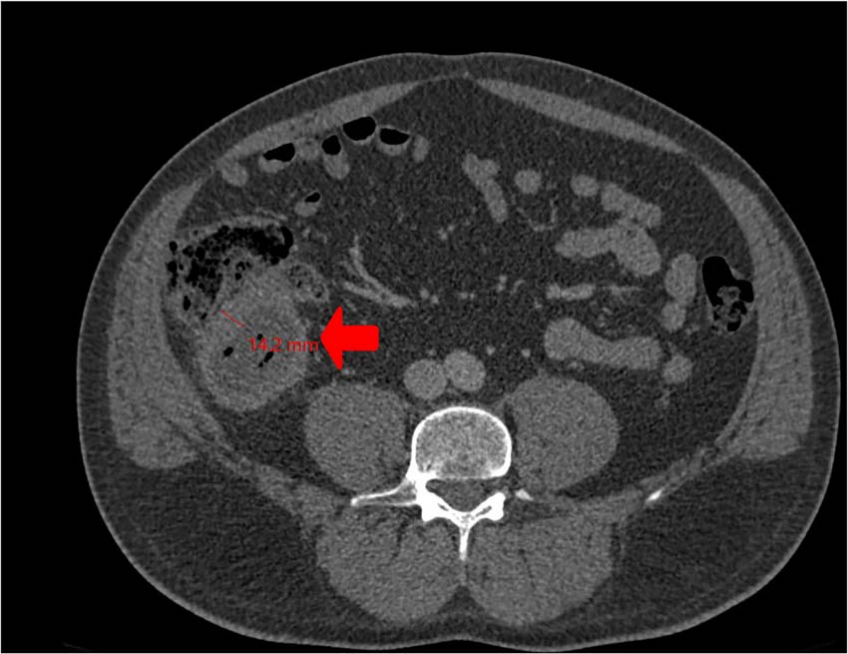
Cross-sectional CT view shows the mass measuring 13 × 5.2 cm with significant wall thickening measuring 14.2 mm and fat stranding. The contents are a mixture of fluid and gas, consistent with appendicitis.

**Figure 3:**
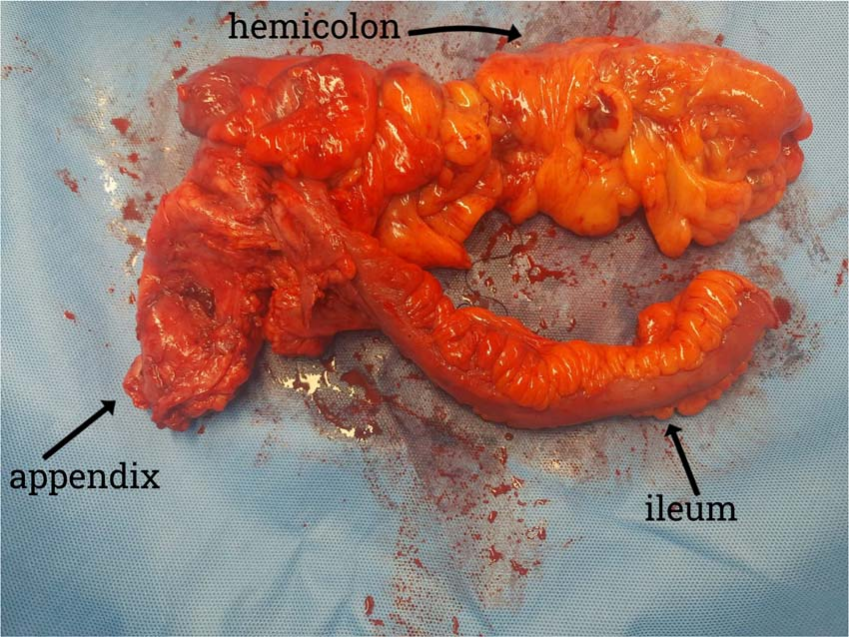
Gross specimen showing the terminal ileum, cecum, appendix, and ascending colon. The appendix is visible near the base of the cecum.

A CT scan showed an oval, 13 × 5.2 cm mass in the right iliac fossa arising from the cecum and running upward, with a thick, edematous wall, fluid, gaseous content, and extensive fat stranding. There was no free fluid and no dilation of bowel, suggesting appendicitis (Figs 1 and 2). Due to these findings, the patient underwent an emergency laparoscopic appendectomy that was converted to open laparotomy. The patient ended up with a right hemicolectomy instead of appendectomy as the appendix could not be separated from the mass, with no separation line between the mass and the cecum. The mass (Fig. 3) measured 10 × 7 × 7 cm and had a thick wall and pyogenic membrane.

Pathology showed a moderately differentiated MAA (G2), in the distal part of the appendix, infiltrating muscularis mucosa into the subserosa without reaching the serosa. All regional lymph nodes were negative for tumor, resulting in a pTNM Classification of pT_3_N_0_M_0_ using the American Joint Committee on Cancer (AJCC) 9^th^ version. The surgical margins and the base of the appendix were free of tumor. Tumor marker levels were within normal limits (Table 1). Postoperatively, the patient recovered well and was discharged on Day six.

**Table 1:** Results of biomarkers test (CA-125, CA-19.9)

Tumor marker	Result value	Reference range
CA-125	29.64 u/ml	0–35 u/ml
CA-19.9	6.71 u/ml	0–37 u/ml

## Discussion

Primary neoplasms of the appendix are rare with an incidence of 0.12 cases per 100 000 people per year [1]. They are found unexpectedly in ~1% of all appendectomy specimens, and although rare, their incidence is increasing [1] They may arise from an epithelial source of origin or have a nonepithelial origin. Two-thirds of these cases are adenocarcinomas [2], classified into four histologic subtypes: signet-ring cell, goblet-cell, mucinous, and nonmucinous (colonic) [4]. MAA is the most common histologic subtype, often presenting at 60 [2]. Primary appendiceal neoplasms typically present as acute appendicitis [5], as was seen in this case. This occurs due to mucoceles obstructing the appendix, leading to cystic dilation. Patients might also present with intestinal obstruction, mucin-filled hernia, genitourinary symptoms [6], incidentally, or with increasing abdominal girth, secondary to pseudomyxoma peritonei (PMP) [7]. PMP develops due to the rupture of the mucus-secreting tumor, with mucin and tumor cells disseminating throughout the peritoneal cavity [8, 9]. Patients with MAA often present after the rupture of the primary tumor, leading to the spread of mucin and tumor cells in the peritoneal cavity [10]. However, if detected before rupture, as in our case (although incidentally), there is potential for improved patient outcomes.

Our patient initially presented with symptoms resembling appendicitis, which was our primary suspicion. A CT abdomen was done to confirm the diagnosis and an emergency laparoscopic appendectomy was planned. CT is the gold standard for appendiceal adenocarcinoma, offering better anatomical topography than ultrasonography (US), including the ability to distinguish between the cecum and mucocele. Preoperative contrast-enhanced CT has a sensitivity of 95% for detecting appendiceal tumors in patients with symptoms of appendicitis [11]. However, our patient was among the 5% where the CT scan did not detect the tumor. Although both magnetic resonance imaging (MRI) and CT are acceptable for preoperative assessment [10], MRI could potentially provide more accurate assessments of the extent of peritoneal disease compared to CT scans [12]. During laparoscopy, an adherent mass was identified at the cecum with an appendiceal origin. As a result, the procedure was converted to a laparotomy for resection and evaluation of the suspected neoplasm. Cystic dilatation of the appendix can be seen in CT scans. An appendiceal diameter greater than 15 mm is not specific but should raise suspicion of malignancy. In this case, the patient's appendix wall thickness measured 14.2 mm. The confirmatory diagnosis is made postoperatively on histopathology, which shows abundant extracellular mucin with low-grade or high-grade nuclear atypia depending on tumor grade. In our report, histology revealed a moderately differentiated MAA.

For staging and follow-up, MRI is recommended for higher sensitivity and specificity to detect peritoneal dissemination [12]. Laboratory findings are nonspecific and might show anemia or elevated tumor markers [13], though our patient’s results were normal. The optimal treatment for appendiceal neoplasms is surgical resection. Laparotomy is recommended to remove an intact specimen because a disrupted specimen may result in PMP [14, 15]. Right colon resection was done in our case.

Ongoing follow-up is essential. Depending on the extent of the disease and the completeness of the resection, some patients may benefit from heated intraperitoneal chemotherapy [14]. Prognosis is generally favorable when the disease is confined to the appendix and resected without rupture [12]. Reporting this case is important since half of appendiceal neoplasms present initially as acute appendicitis [9]. Further research is needed to better understand MAA and improve treatment plans.
